# The Oleates

**Published:** 1885-08

**Authors:** 


The Oleates: an Investigation into their Nature and Action. By John V. Shoemaker, A. M., M. D., Lecturer on Dermatology at the Jefferson Medical College; Physician to the Philadelphia Hospital for Skin Diseases; Member of the Pennsylvania State Medical Society; The Minnesota State Medical Society; The American Medical Association; The American Academy of Medicine; The British Medical Association; Fellow of the Medical Society of London, etc., etc. Philadelphia: F. A. Davis, publisher. 1885.

This little book is a resumé of Dr. Shoemaker's valuable papers that have already been published in the different medical journals and in the Transactions of the Pennsylvania State Medical Society, with several chapters of new matter never before published. The book contains much that is valuable.

